# Synergistic Integration of Carbon Quantum Dots in Biopolymer Matrices: An Overview of Current Advancements in Antioxidant and Antimicrobial Active Packaging

**DOI:** 10.3390/molecules29215138

**Published:** 2024-10-30

**Authors:** Ajit Kumar Singh, Pontree Itkor, Myungho Lee, Aphisit Saenjaiban, Youn Suk Lee

**Affiliations:** 1 Department of Packaging & Logistics, Yonsei University, Wonju 26393, Republic of Korea; ajitksingh@yonsei.ac.kr (A.K.S.); pontree.itkor@yonsei.ac.kr (P.I.); ho_mung2@yonsei.ac.kr (M.L.); 2 Doctor of Philosophy Program in Nanoscience and Nanotechnology (International Program/Interdisciplinary), Faculty of Science, Chiang Mai University, Chiang Mai 50200, Thailand; aphisit_saen@cmu.ac.th

**Keywords:** active packaging, carbon quantum dots, biopolymers, antioxidants, antimicrobials, food safety, sustainability

## Abstract

Approximately one-third of the world’s food production, i.e., 1.43 billion tons, is wasted annually, resulting in economic losses of nearly USD 940 billion and undermining food system sustainability. This waste depletes resources, contributes to greenhouse gas emissions, and negatively affects food security and prices. Although traditional packaging preserves food quality, it cannot satisfy the demands of extended shelf life, safety, and sustainability. Consequently, active packaging using biopolymer matrices containing antioxidants and antimicrobials is a promising solution. This review examines the current advancements in the integration of carbon quantum dots (CQDs) into biopolymer-based active packaging, focusing on their antioxidant and antimicrobial properties. CQDs provide unique advantages over traditional nanoparticles and natural compounds, including high biocompatibility, tunable surface functionality, and environmental sustainability. This review explores the mechanisms through which CQDs impart antioxidant and antimicrobial activities, their synthesis methods, and their functionalization to optimize the efficacy of biopolymer matrices. Recent studies have highlighted that CQD-enhanced biopolymers maintain biodegradability with enhanced antioxidant and antimicrobial functions. Additionally, potential challenges, such as toxicity, regulatory considerations, and scalability are discussed, offering insights into future research directions and industrial applications. This review demonstrates the potential of CQD-incorporated biopolymer matrices to transform active packaging, aligning with sustainability goals and advancing food preservation technologies.

## 1. Introduction

**Figure 1:**
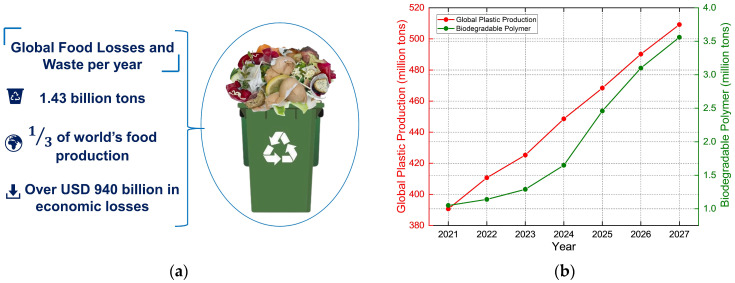
(**a**) Worldwide scenario of food losses and waste; (**b**) projected global plastic production and biopolymer production 2021–2027.

The global food system faces unprecedented challenges in ensuring sustainability and food security and in reducing economic losses owing to food waste. The Food and Agriculture Organization (FAO) reports that approximately one-third of the global food production, approximately 1.43 billion tons, is wasted each year, resulting in economic losses of nearly USD 940 billion (Figure 1a) [1]. This level of waste exhausts key resources, including water, land, energy, labor, and capital, and intensifies environmental challenges, particularly the release of greenhouse gases that drive climate change. Moreover, food waste increases the price of food and compromises food security, further complicating efforts to feed a growing global population [2,3]. On a larger scale, food packaging plays a vital role in preserving food quality and extending shelf life. However, traditional packaging falls short of addressing modern market demands for enhanced food safety, prolonged shelf life, reduced food waste, and sustainable packaging solutions. The primary concern regarding traditional plastics derived from petroleum sources is the significant amount of mismanaged waste, which is rapidly increasing. Packaging is a major area of concern because of its single-use nature, consumer behavior, and high production and consumption rates, leading to substantial waste accumulation. The amount of plastic produced globally was 390.7 million tons in 2021, and by 2027, production is projected to exceed 500 million tons. This dramatic increase highlights the urgent need for essential measures to address plastic waste management and reduce its environmental impact. Consumers and industry stakeholders are increasingly seeking packaging materials that not only protect food but also contribute to environmental sustainability and public health [4,5,6]. As shown in Figure 1b, after stagnation in 2021–2022, primarily owing to the COVID-19 pandemic, global biodegradable polymer production resumed its growth in 2023. Biodegradable polymer production is expected to expand significantly, with its capacity increasing from approximately 1.05 million tons in 2021 to an estimated 3.56 million tons by 2027. Biodegradable polymers such as polylactic acid (PLA), polyhydroxyalkanoates (PHA), starch-based polymers, and cellulose are among the most widely produced and utilized materials in sustainable packaging [6]. These polymers are favored due to their biodegradability and potential to reduce the environmental impact of packaging waste. The increasing adoption of these materials indicates the shift towards eco-friendly packaging solutions that meet both environmental and functional requirements [5,6].

Active packaging has been identified as a promising and innovative solution to address these challenges. It directly incorporates functional agents, such as antioxidants and antimicrobials, into packaging materials to actively control and prolong the freshness and safety of food products [7,8]. Commonly used antioxidants include butylated hydroxytoluene (BHT), ascorbic acid, tocopherols, and citric acid [9]. These antioxidants help prevent food spoilage by neutralizing free radicals, which are unstable molecules that can cause oxidative damage to lipids, proteins, and DNA in food products [10,11]. Antioxidants inhibit the oxidation process by scavenging free radicals, thereby preserving the color, flavor, and nutritional quality of food. For instance, Castro et al. (2019) demonstrated that the inclusion of natural antioxidants, such as green tea extract in whey protein packaging films, significantly reduces lipid oxidation in packaged salmon, thereby extending its shelf life [12]. On the other hand, antimicrobials inhibit the growth of microorganisms by disrupting microbial cell walls, inhibiting cell division, or interfering with microbial metabolism, thereby enhancing the safety and extending the shelf life of food products [13]. Examples of antimicrobials include silver nanoparticles, chitosan, nisin, and triclosan, which are incorporated into packaging materials to create hostile environments for bacterial growth [14,15,16,17]. Méndez et al. (2023) demonstrated the effectiveness of chitosan-/gelatin-based films combined with eugenol and oregano essential oil in reducing the microbial load on fresh cheese [18]. Among the various materials explored for active packaging, biopolymers have garnered significant attention because of their biodegradability and minimal environmental impact [19]. Commonly used biopolymers, such as polylactic acid, polyhydroxyalkanoates, starch-based polymers, cellulose, chitosan, and alginate, offer sustainable alternatives to traditional plastics. However, to satisfy the stringent functional demands of active packaging applications, biopolymers often require an enhancement in their inherent properties [20,21].

Carbon quantum dots (CQDs), a class of zero-dimensional nanomaterials, have recently garnered significant interest owing to their potential applications in active packaging. CQDs exhibit unique properties, including high biocompatibility, tunable surface functionalities, excellent antioxidant and antimicrobial activities, and environmental sustainability [22,23,24,25]. Unlike traditional nanoparticles and natural compounds, CQDs can be precisely engineered to satisfy specific functional requirements, which makes them highly versatile for use in food-packaging applications. Their ability to scavenge free radicals and inhibit microbial growth makes them suitable for applications aimed at preserving food quality and safety. The antioxidant mechanism of CQDs primarily involves their ability to neutralize free radicals, which are unstable molecules that cause oxidative damage to lipids, proteins, and DNA in food products [26,27]. Unlike traditional antioxidants, CQDs possess a large surface-area-to-volume ratio and abundant functional groups, such as carboxyl and hydroxyl groups, which enhance their electron-donating capabilities [28]. This allows CQDs to effectively scavenge free radicals, thereby preventing oxidative processes that lead to food spoilage. Several studies have demonstrated that CQDs can be engineered to possess superior antioxidant activities compared with natural antioxidants, such as ascorbic acid and tocopherols, owing to their tunable surface functionalities and photoluminescent properties [29,30]. Moreover, the ability of CQDs to generate reactive oxygen species (ROS) upon exposure to light irradiation is linked to their antimicrobial properties. ROS can damage microbial cell walls, proteins, and nucleic acids, eventually resulting in cell death. The small size and high surface charge of CQDs enable them to interact closely with microbial cell membranes, causing physical disruptions and enhancing their antimicrobial efficacy [31,32]. Additionally, CQDs can be functionalized with various antimicrobial agents, such as essential oils or natural nanoparticles, to enhance their activity [33]. This multifunctionality makes CQDs more versatile and effective than traditional nanoparticles or natural antimicrobial compounds. For example, although silver nanoparticles are effective antimicrobials, there exist concerns regarding their cytotoxicity and environmental impact, which are significantly reduced with the use of biocompatible CQDs [34,35,36].

Considering the significance of CQDs in enhancing biopolymer-based active packaging, this review provides an overview of recent advancements, focusing on their antioxidant and antimicrobial properties. It explores the mechanisms through which these nanomaterials improve food preservation and discusses synthesis and functionalization methods to optimize their performance. Additionally, key challenges such as toxicity, regulatory hurdles, and scalability are addressed, with insights into future research and industrial applications. By summarizing the latest developments and identifying critical areas for exploration, this review highlights the potential of CQD-incorporated biopolymers to advance packaging systems, promote sustainability, and enhance food preservation.

## 2. Carbon Quantum Dots: Overview, Structural Chemistry, and Synthesis Routes

### 2.1. Overview

**Figure 2:**
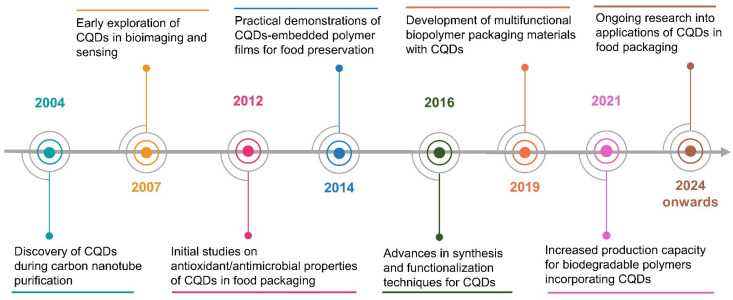
Timeline of key developments in CQDs and their applications in food packaging.

In recent years, CQDs have emerged as a novel class of zero-dimensional nanomaterials, characterized by their small size (typically <10 nm), unique optical and electronic properties, high biocompatibility, and sustainability [37,38]. Unlike traditional nanoparticles and natural compounds used in food packaging, CQDs offer a combination of functional properties, including antioxidant and antimicrobial activities, low toxicity, and environmental friendliness, making them particularly suitable for enhancing the characteristics of biopolymer matrices in sustainable packaging solutions [31,39]. CQDs were discovered incidentally during the purification of single-walled carbon nanotubes in 2004 [40]. This discovery marked the beginning of interest in CQDs owing to their unique optical properties, including strong photoluminescence (PL) and low toxicity, compared with traditional semiconductor quantum dots. Figure 2 illustrates the timeline of the key developments in CQDs since 2004. Initial studies have focused on understanding the fundamental properties of CQDs, including their size-dependent optical behavior, surface chemistry, and biocompatibility. These properties have led to primary applications in bioimaging and sensing owing to the excellent fluorescence properties of CQDs. The recognition of other potential characteristics has facilitated the application of CQDs in food packaging [33,41,42].

Significant advancements have been made in the synthesis and functionalization of CQDs. Methods, such as hydrothermal/solvothermal, microwave-assisted, and pyrolytic syntheses, have been optimized to produce CQDs with tailored properties for specific applications in food packaging. These advancements have enabled the production of CQDs with controlled sizes, shapes, and surface functional groups, enhancing their integration into biopolymer matrices [30,43]. Since their discovery, the structure, synthesis methods, and various properties of CQDs have been comprehensively studied by researchers in numerous fields, such as bioimaging, sensing, solar energy, environmental science, drug delivery, photocatalysis, optoelectronics, agriculture, and food packaging [44,45,46,47,48,49,50,51]. The integration of CQDs into food-packaging materials represents a significant advancement in the development of sustainable and functional packaging solutions. Recent studies have demonstrated that CQDs hold considerable promise in extending the shelf life of food products and reducing waste, thereby aligning with global sustainability goals [52,53].

### 2.2. Structural Chemistry of CQDs

**Figure 3:**
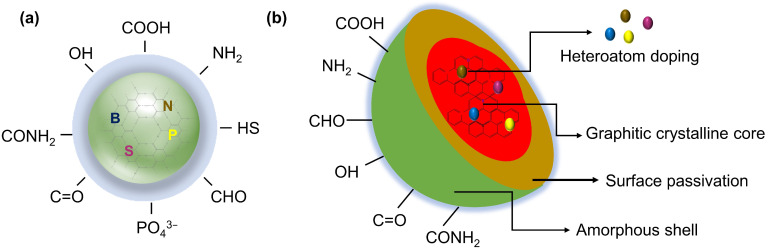
Structural overview of CQDs: (**a**) generalized structure of CQDs; (**b**) detailed illustration of the core–shell structure of CQDs.

CQDs exhibit a distinct core–shell structure formed through a nucleation process that involves the gradual growth of a core, followed by the development of a self-passivated shell composed of various functional groups [54]. This nucleation process begins with the initial formation of small carbon clusters, which gradually coalesce to form the cores of the CQDs. Subsequently, the functional groups attached to the surface of these cores form a shell, as shown in Figure 3a. The core of CQDs, also known as the intrinsic state, can be either graphitic crystalline (sp^2^) or amorphous (a mixture of sp^2^ and sp^3^ hybridized carbons), depending on the degree of sp^2^ carbon present [55,56]. Furthermore, the nature of the core is significantly influenced by the synthesis technique, precursors used, and specific synthesis conditions, such as temperature, time, and pH [57]. Several researchers have reported that the cores of CQDs are typically graphitic crystalline (sp^2^) and are characterized by a well-defined lattice structure, as shown in Figure 3b [37,58]. These graphitic cores are usually small, ranging between 2 and 3 nm in size, with a typical lattice spacing of approximately 0.2 nm [54]. A high degree of graphitization (sp^2^ carbon) within the core is often associated with enhanced photoluminescent properties and better chemical stability, making these CQDs particularly suitable for applications requiring strong fluorescence and robust performance under various environmental conditions [59]. The synthesis technique and conditions also play a vital role in determining the presence and distribution of functional groups on the surface of CQDs. Functional groups, such as hydroxyl (–OH), carboxyl (–COOH), and amino (–NH_2_) groups, form self-passivated shells around the core [60]. This shell enhances the solubility and stability of the CQDs in different solvents, imparting specific chemical functionalities that can be modified for various applications. For instance, in food packaging, the hydroxyl (–OH) and carboxyl (–COOH) groups on CQDs enhance their hydrophilicity, facilitating their integration into biopolymer matrices [43]. This integration is vital for maintaining the antioxidant properties of the packaging by neutralizing free radicals and preventing oxidative spoilage. Additionally, amino (–NH_2_) groups enable further functionalization, enabling the conjugation of antimicrobial agents that can inhibit microbial growth, thereby extending the shelf life and safety of food products [61,62].

### 2.3. Synthesis of CQDs

Several synthesis routes encompassing both physical and chemical methodologies have been developed to facilitate the fabrication of CQDs [55]. In the context of sustainable packaging, there is pronounced emphasis on integrating environmentally benign and sustainable manufacturing processes. These processes are significant because they exert a considerable influence on the surface functionalities and characteristics of the CQDs, which are effective in determining their physicochemical properties and subsequent interactions with biological interfaces. These strategies not only enable the predictable and consistent production of CQDs with remarkable optical and functional properties but also align with the principles of green chemistry. The production methods of CQDs are typically divided into “top-down” and “bottom-up” approaches, with the choice of approach contingent upon the interaction dynamics between the raw materials and the specific procedures employed [33,55].

The top-down approach involves breaking down larger carbon structures, such as graphite, graphene, or carbon nanotubes, into smaller fragments through processes such as laser ablation, arc discharge, chemical oxidation, and electrochemical synthesis [63]. These methods often yield CQDs containing a mixture of sp^2^ and sp^3^ hybridized carbon atoms, reflecting the diverse nature of the starting materials. However, these top–down methods can be energy-intensive, and controlling the size and uniformity of CQDs can be challenging, making them less favorable for large-scale production [55,64]. In contrast, the bottom–up approaches involve the synthesis of CQDs from smaller molecular precursors through chemical reactions, making them more suitable for large-scale production. These methods adhere to green chemistry principles because they are simple, cost-effective, and energy-efficient. The bottom–up synthesis methods include hydrothermal/solvothermal synthesis, microwave-assisted synthesis, and pyrolysis, with the hydrothermal synthesis route being particularly preferred owing to its simplicity and environmental friendliness [65]. This method involves the reaction of carbon-rich precursors in an aqueous medium at elevated temperatures and pressures, leading to the formation of CQDs with distinct surface functionalities and controlled sizes [66]. Common carbon-rich precursors used for CQDs synthesis include a variety of organic materials, such as citric acid, glucose, and biomass-derived materials like fruit peels, coffee grounds, and other agricultural residues [50,53]. These renewable and abundant sources make CQDs synthesis both environmentally friendly and cost-effective, further aligning with the goals of sustainable packaging. Following the synthesis of CQDs, isolation and purification are necessary to remove unreacted precursors and byproducts. The size of CQDs synthesized through these methods typically falls within the range of 1 to 10 nm, although the exact size can vary depending on the specific precursor materials and synthesis conditions used [55]. Common purification techniques include centrifugation, which separates CQDs based on size and density; dialysis, which removes small molecules and salts using a semipermeable membrane; and gel electrophoresis, which separates CQDs based on size and charge [67,68]. These methods ensure that the final product is of high purity and is suitable for various applications, including bioimaging, sensing, and food packaging.

## 3. Characterization of CQDs

**Figure 4:**
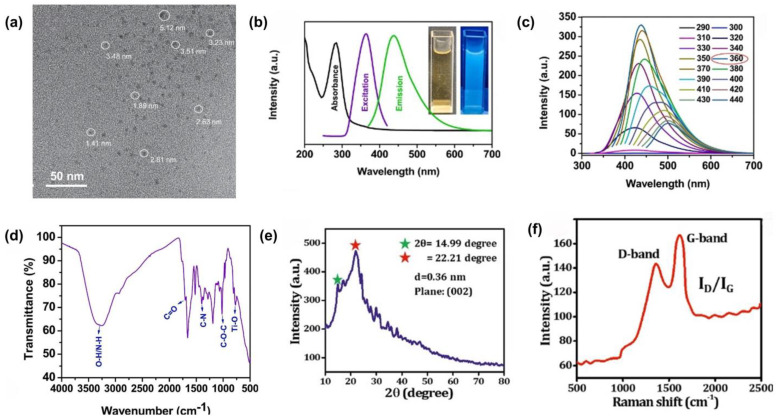
Characterization of CQDs. (**a**) TEM image of glucose-derived CQDs; (**b**) UV–vis, PL emission, and excitation spectra; (**c**) PL emission spectra; (**d**) FTIR spectra of TiO_2_-doped CQDs; (**e**) XRD pattern; (**f**) Raman spectrum of CQDs. Reproduced with permission from (**a**) [72]; (**b**–**d**) [73]; (**e**,**f**) [51].

Following the synthesis of CQDs, their detailed characterization is essential to systematically recognize and optimize their potential applications. Characterization typically involves analyzing the morphological, optical, and structural properties of the CQDs, as these attributes are fundamental in determining their functional characteristics. The structure of CQDs is primarily influenced by the raw materials and synthesis methods, which significantly affect their formation, reaction conditions, and final properties [69]. These factors collectively contribute to the effectiveness of CQDs in enhancing the safety and extending the shelf life of packaged food. The morphological features of CQDs are typically examined using transmission electron microscopy (TEM), which provides high-resolution images to determine the size, shape, and distribution of the quantum dots. For sustainable packaging applications, CQDs must maintain a uniform and small size, typically <10 nm, to ensure an optimal surface area for interaction with biopolymer matrices [70]. The uniform size also contributes to consistent PL properties and reactivity, both of which are essential for applications such as active and intelligent packaging [71]. In addition, TEM can reveal the degree of crystallinity within the CQDs, where a more graphitic structure is often associated with enhanced stability and photoluminescent efficiency, which are important factors for the durability and functionality of packaging materials. For instance, Figure 4a shows a TEM image of CQDs derived from glucose, ranging from 1.4 to 5.1 nm in size, with an average diameter of 3.52 nm [72]. Sandeep et al. reported CQDs with spherical, homogeneously monodispersed particles in the size range of 5–7 nm. In their investigation, high-resolution TEM images revealed distinct lattice fringes with a spacing of 0.22 nm, corresponding to the (002) plane characteristic of graphene [51].

The optical properties of CQDs are another essential aspect of their characterization, particularly their PL behavior, which is of considerable interest for applications in bioimaging, sensing, and active packaging [39,74]. Ultraviolet–visible (UV–vis) spectroscopy is commonly used to examine the absorption spectra of CQDs, typically displaying characteristic absorption peaks corresponding to the π–π* transitions of the conjugated carbon network and the n–π* transitions of surface functional groups [75]. For example, the UV–vis spectrum of TiO_2_-doped CQDs derived from sweet potato peel showed a sharp band at 285 nm, indicating the presence of the C=O group (n-π* transition), as illustrated in Figure 4b [73]. These TiO_2_-doped CQDs, which appeared as brownish solutions in sunlight, exhibited high PL emission with noticeable blue fluorescence under UV light (365 nm), as shown in the inset of Figure 4b. In addition, the PL emission spectra of the CQDs, which can be finely tuned across the visible spectrum by altering their size and surface functional groups, are particularly valuable for the development of intelligent packaging systems. These systems can provide visual indicators of food freshness or spoilage by emitting specific colors in response to environmental changes, such as shifts in pH or the presence of gases, thereby enhancing food safety and consumer awareness. PL spectroscopy is also widely used to study the emission properties of CQDs, revealing information on the quantum yield, emission wavelength, and effects of surface passivation [76]. In a study by Riahi et al. focusing on carrageenan-based sustainably active and pH-dependent color-changing composite films, the PL emission spectra demonstrated that TiO_2_-doped CQDs exhibited an excitation-dependent phenomenon with an apparent red shift when excited at longer wavelengths [73]. These CQDs display a maximum fluorescence emission peak at 437 nm under an optimal excitation wavelength of 360 nm, as shown in Figure 4c. The tunable PL emission of CQDs is influenced by several factors, including carbon sources, changes in size and shape, and the presence of distinct luminescent sites on their surface.

Furthermore, structural characterization techniques, such as Fourier-transform infrared (FTIR) spectroscopy, X-ray diffraction (XRD), and Raman spectroscopy, were employed to determine the chemical composition, bonding environment, and crystalline structure of the CQDs. FTIR spectroscopy was used to identify the functional groups present on the surface of CQDs, such as hydroxyl (–OH), carboxyl (–COOH), and amino (–NH_2_) groups, which are essential for their functional properties, such as antioxidant and antibacterial activities in food packaging [33,43]. The FTIR spectrum of the TiO_2_-doped CQDs, shown in Figure 4d, confirmed the abundance of –OH, –COOH, and –NH_2_ groups in the synthesized CQDs [73]. XRD analysis provided insights into the crystalline structure of the CQDs, differentiating between amorphous and graphitic regions. As shown in Figure 4e, the XRD pattern of the CQDs derived from expanded polystyrene foam via the solvothermal approach indicated a mixed structure with both amorphous carbon and graphitic crystalline regions [51]. The presence of the (002) plane suggests that localized graphitic structures exist within the CQDs despite their predominantly amorphous nature. Raman spectroscopy complements XRD by providing detailed information regarding the sp^2^/sp^3^ carbon hybridization ratio. Generally, CQDs exhibit two characteristic bands in Raman spectroscopy: the D band and the G band. The D band, typically observed around 1350 cm^−1^, is associated with structural defects or disorder in the carbon framework, while the G band, found around 1580 cm^−1^, corresponds to graphitic order, representing sp^2^-hybridized carbon atoms. The intensity ratio of these bands (I_D_/I_G_) is commonly used to assess the degree of disorder and crystallinity in carbon-based materials, including CQDs [55]. In Figure 4f, the Raman spectrum of CQDs derived from expanded polystyrene foam shows two peaks at 1362 and 1605 cm^−1^, corresponding to the D and G bands, respectively. The I_D_/I_G_ band intensity ratio (2.12) was used to quantify the sp^3^-hybridized carbon atoms and assess the defect and disorder densities within the CQDs [51].

## 4. Functional Properties of CQDs

The functional properties of nanomaterials have garnered significant attention because of their potential to enhance safety and extend shelf life in advanced food-packaging applications. Among these, CQDs have emerged as novel functional nanomaterials with exceptional versatility, particularly for their roles as antioxidants and antimicrobials. The strong antioxidant and antimicrobial activities of CQDs are primarily driven by their unique structural characteristics, such as large surface area, abundant functional groups, and tunable PL. These properties enable CQDs to play a vital role in extending the shelf life of food products by preventing oxidative damage and inhibiting microbial growth, which are the primary causes of food spoilage and contamination. In this study, the antioxidant and antimicrobial properties of CQDs were explored in detail with a focus on their underlying principles, mechanisms of action, and practical applications in food packaging.

### 4.1. Antioxidant Properties

Oxidation is a significant factor contributing to food spoilage, with estimates suggesting that approximately 20–25% of global food waste is directly attributed to oxidation-mediated degradation [77,78]. Oxidation not only leads to substantial economic losses but also intensifies food insecurity by reducing the quality and safety of food products before they reach consumers [79]. Addressing these challenges has become a focal point in recent studies, which have increasingly explored innovative strategies to prevent the deterioration of food quality caused by oxidative reactions. One of the most promising approaches involves the incorporation of antioxidants into packaging materials, edible films, and coatings. Over the past few decades, synthetic antioxidants, such as butylated hydroxyanisole (BHA), BHT, and tert-butyl hydroquinone, have been extensively used in the food industry because of their effectiveness and cost efficiency [80]. These compounds are particularly effective at inhibiting lipid oxidation, which is the primary cause of rancidity in fats and oils. For example, BHT and BHA are commonly used in packaged snack foods, cereals, and cooking oils to extend their shelf life by preventing oxidative rancidity [81]. However, the potential health risks associated with synthetic antioxidants have raised concerns, including studies suggesting their possible carcinogenic or toxic effects, leading to increased regulatory scrutiny and consumer apprehension [82]. Consequently, there has been a growing shift toward natural antioxidants derived from plant-based sources, which are perceived as safer and more in line with the demand for clean-label products [83,84]. Natural antioxidants include polyphenols, carotenoids, vitamins (such as vitamins C and E), and essential oils, typically extracted from fruits, vegetables, herbs, and other natural sources [8]. However, the challenges associated with natural antioxidants include variability in activity based on the source and extraction method, potential interactions with other food components, and sometimes higher costs than synthetic alternatives. Moreover, natural antioxidants have limited stability and effectiveness over prolonged storage periods, which can affect their performance in food-packaging applications [13].

**Figure 5:**
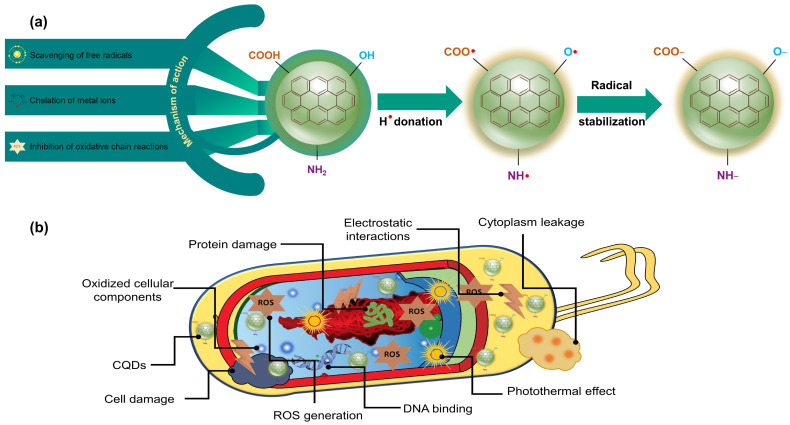
Mechanistic illustration of the functionalities of CQDs: (**a**) antioxidant activity through free radical scavenging, metal ion chelation, and inhibition of oxidative chain reactions; (**b**) antimicrobial mechanism involving ROS generation, electrostatic interactions, and membrane disruption.

In this context, CQDs represent a novel class of nanomaterials with potent antioxidant properties and offer a promising approach for food preservation. The antioxidant activities of CQDs are closely linked to their unique structural features, including large surface area, nanoscale size, and the presence of various functional groups, such as –OH, –COOH, and –NH_2_ groups [85]. These functional groups play a key role in enabling CQDs to scavenge ROS, which are the primary agents responsible for oxidative spoilage in food products. The mechanism of the antioxidant action of CQDs, as illustrated in Figure 5a, involves several pathways. First, CQDs directly interact with and neutralize free radicals by donating hydrogen atoms or electrons, thereby preventing the initiation of oxidative chain reactions that can degrade food quality [86]. Second, CQDs can chelate metal ions, such as Fe^2+^ and Cu^2+^, which catalyze the formation of ROS. By binding to these metal ions, CQDs effectively inhibit further ROS generation, reducing the overall oxidative stress in food [26,87]. In addition, CQDs can interrupt oxidative chain reactions by stabilizing intermediate radicals and halting the propagation of oxidative damage [88].

The incorporation of CQDs into biopolymer-based packaging materials represents a significant advancement in active packaging systems designed to extend the shelf life of food products while preserving their nutritional and sensory attributes. Numerous studies have demonstrated that CQD-incorporated films can significantly reduce oxidative rancidity and maintain the quality of sensitive nutrients, making them compelling alternatives to traditional synthetic antioxidants [43,86,89,90,91]. Furthermore, the use of CQDs is aligned with the growing trend toward sustainability and natural food preservation, providing a biocompatible and environmentally friendly solution to the challenges of food spoilage and waste. For instance, Yang et al. synthesized CQDs from coffee husks and incorporated them into carboxymethyl cellulose (CMC)-based active composite films, which demonstrated strong antioxidant properties, achieving an effectiveness of more than 95% in 1,1-diphenyl-2-picrylhydrazyl (DPPH) and 2,2-azino-bis-3-ethylbenzothiazoline-6-sulphonic acid (ABTS) assays [52]. In food-packaging tests, these films significantly slowed the decay of fresh-cut apples, extending their shelf life to 7 d under refrigerated storage at 4 °C. Another study highlighted the enhancement in antioxidant activity in CQDs derived from *Pseudomonas aeruginosa*, which were further doped with sulfur [92]. S-doped CQDs exhibited a remarkable increase in antioxidant efficacy, achieving scavenging rates of 94.98% for ABTS radicals and 83.63% for DPPH radicals. When incorporated into polyvinyl alcohol (PVA)-based films, the S-doped CQDs demonstrated a significant potential for extending the shelf life of packaged pork meat and sliced apples by preventing lipid oxidation and inhibiting browning, respectively.

### 4.2. Antimicrobial Activity

The incorporation of antimicrobial agents into biopolymer matrices has become a widely adopted strategy for developing food-packaging films and coatings with antibacterial properties. Traditionally, inorganic nanoparticles, such as silver, zinc oxide, and titanium dioxide, have been extensively used because of their effective antimicrobial properties [93,94]. These nanoparticles typically release metal ions that penetrate microbial cell membranes, generate ROS, and disrupt microbial DNA, ultimately leading to cell death [95]. Despite their effectiveness, concerns have been raised regarding the potential toxicity of these inorganic additives, particularly their long-term effects on human health and the environment. This has driven a shift toward the exploration of more sustainable and natural alternatives. Natural antibacterial agents, including plant extracts, organic acids, polyphenols, and essential oils, have gained prominence as safer options for imparting antibacterial properties to food-packaging materials [8]. Natural compounds, which are often derived from fruits, vegetables, and herbs, are directly incorporated into the film matrix to inhibit bacterial growth. Although these agents offer a safer profile, their efficacy can vary, and they may present challenges in terms of stability, effectiveness over time, and interactions with other food components [96].

In addition to these natural agents, CQDs have emerged as promising sustainable and nontoxic antibacterial nanoparticles. CQDs offer several advantages over traditional antimicrobial agents, including photostability, ease of surface functionalization, and biocompatibility, which enhance their interactions with bacterial cells [97]. Furthermore, CQDs can be synthesized from abundant, low-cost, and nontoxic precursors, making their production both economical and environmentally friendly. The nanoscale size of CQDs, combined with their large surface area, biocompatibility, and water solubility, facilitates close contact with microbial surfaces, thereby enhancing their antibacterial efficacy. Recent studies have demonstrated the broad-spectrum antimicrobial activity of CQDs against various microorganisms, including bacteria, fungi, and viruses, making them suitable for applications in food packaging. As shown in Figure 5b, the antimicrobial mechanism of CQDs is multifaceted and distinct from that of conventional antibiotics, involving both chemical and physical interactions with microbial cells. A primary mechanism is the generation of ROS, which include highly reactive chemical species, such as hydroxyl radicals (OH^•^), singlet oxygen (^1^O_2_), superoxide anions (O_2_^•−^), and hydrogen peroxide (H_2_O_2_) [98]. These ROS induce oxidative stress in microbial cells, leading to damage to cellular components, such as lipids, proteins, and DNA, ultimately resulting in cell death. Furthermore, CQDs can physically interact with microbial cell membranes, causing structural disruption and leakage of intracellular contents, leading to the degeneration of the cell structure and the leakage of the cytoplasm. Additionally, CQDs can bind to microbial DNA, modulate gene expression, and contribute to cell death [98,99].

**Figure 6:**
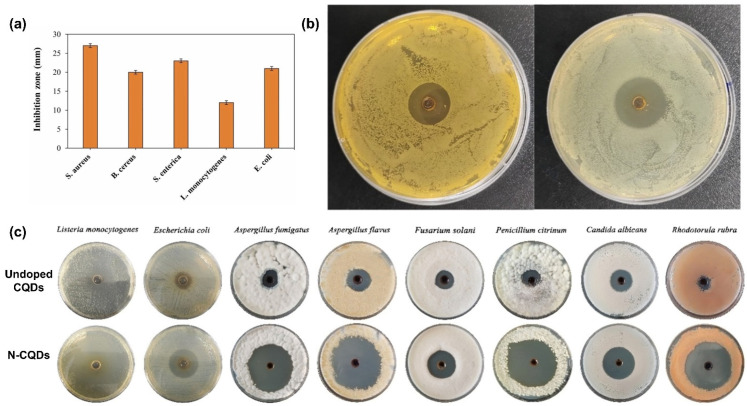
Antimicrobial activity of CQDs. (**a**) Antimicrobial efficacy of lemon-peel-derived CQDs against various bacterial strains; (**b**) inhibition zones formed by nitrogen-doped CQDs (N-CQDs) synthesized with ethylenediamine against *E. coli* and *L. monocytogenes*; (**c**) comparative antibacterial assay of glucose-derived undoped CQDs and N-CQDs using urea, showing inhibition zones against different bacterial species. Reproduced with permission from (**a**) [89]; (**b**) [101]; (**c**) [102].

Several factors influence the antimicrobial properties of the CQDs incorporated in food-packaging materials. The selection of biomass sources for the synthesis of CQDs is of paramount importance because it can lead to variations in the size, surface charge, and functional groups of the CQDs, which directly influence their antibacterial effectiveness. For example, Yang et al. successfully synthesized CQDs from coffee husk waste by leveraging its richness in bioactive polyphenolic compounds, such as gallic acid, tannic acid, chlorogenic acid, and epicatechin [52]. These CQDs exhibited potent antibacterial activities against *L. monocytogenes* and *E. coli*, demonstrating the significant role of bioactive compounds in enhancing the antimicrobial properties of CQDs. Similarly, Jayakumar et al. synthesized CQDs from dried lemon peel, which is rich in polyphenols (ferulic acid, p-coumaric acid, and sinapic acid), flavonoids (flavanones, flavanols, and flavones), vitamin C, and essential oils [89]. As shown in Figure 6a, CQDs derived from lemon peels show strong antibacterial activity against a broad spectrum of bacteria, including *S. aureus*, *B. cereus*, *S. enterica*, *L. monocytogenes*, and *E. coli*. The active ingredients in lemon peels, such as polyphenols and flavonoids, are likely to contribute to the enhanced antimicrobial properties of these CQDs. Furthermore, the synthesis method employed plays a significant role, as it can influence the structure, surface chemistry, and functional properties of the CQDs. For instance, hydrothermal methods may yield CQDs with a high degree of graphitization, thereby enhancing their PL and antimicrobial activities [100].

The surface charge of CQDs is another influencing factor, as it governs the electrostatic interactions between the CQDs and microbial cells. A positively charged surface on CQDs can facilitate stronger electrostatic attraction to the negatively charged bacterial cell membranes, enhancing their antibacterial activity. Bing et al. examined the influence of surface charge on the antibacterial properties of CQDs against *E. coli* [103]. These findings indicate that cationic CQDs exhibit the most potent antibacterial effects, whereas CQDs with anionic and neutral charges are less effective in inhibiting bacterial growth. This study highlights the importance of optimizing the surface charge to maximize the antimicrobial potential of CQDs. Furthermore, the antimicrobial properties of CQDs can be significantly enhanced through functionalization techniques, such as passivation and doping. Doping CQDs with elements, such as nitrogen or sulfur, not only introduces additional functional groups but also alters the electronic structure of the CQDs, which can improve their ability to generate ROS and, consequently, their antimicrobial efficacy. For example, in a study in which CQDs were synthesized hydrothermally using polyethylene glycol (PEG) and nitrogen-doped with ethylenediamine, the modified CQDs exhibited notable antibacterial activity against *E. coli* and *L. monocytogenes* when tested using the agar well diffusion method [101]. The nitrogen-doped CQDs (N-CQDs) produced inhibition zones of up to 14 mm for *E. coli* and 18 mm for *L. monocytogenes*, whereas undoped PEG-based CQDs displayed no significant antimicrobial activity, as illustrated in Figure 6b. The enhanced antibacterial properties of the N-CQDs were attributed to the presence of functional groups such as amides and amines on their surfaces. These functional groups, particularly the protons associated with the amine and amide groups, are believed to facilitate the penetration of N-CQDs into negatively charged bacterial cell walls, thus enhancing their antimicrobial action. Similarly, Ezati et al. studied CQDs synthesized from glucose and subsequently N-doped using urea [102]. This study, as shown in Figure 6c, demonstrated that the antibacterial activity of these CQDs varied significantly between the doped and undoped versions. The N-CQDs exhibited a substantial increase in the inhibition zone against *L. monocytogenes*, from approximately 6 mm for undoped CQDs to approximately 17 mm for N-CQDs. Similarly, the N-CQDs exhibited enhanced antibacterial activity against *E. coli*. Furthermore, in tests against fungal strains, such as *A. fumigatus*, *A. flavus*, *P. citrinum*, *C. albicans*, and *R. rubra*, undoped CQDs produced smaller inhibition zones (4–8 mm), whereas N-CQDs resulted in much larger inhibition zones (10–24 mm).

Furthermore, the bacterial cell wall plays a vital role in resisting the penetration of CQDs into the microbial cells. The structural composition of the cell wall, particularly in Gram-positive and Gram-negative bacteria, significantly influences the extent to which CQDs can interact with and disrupt the cell membrane. Studies using N-CQDs synthesized with ethylenediamine reported inhibition zones of up to 14 mm for *E. coli* (a Gram-negative bacterium) and 18 mm for *L. monocytogenes* (a Gram-positive bacterium) [101]. Similarly, Ezati et al. observed larger inhibition zones for *L. monocytogenes* (17 mm) than for *E. coli* (8 mm). The researchers suggested that the larger inhibition zone observed for *L. monocytogenes* could be due to the higher negative charge of the Gram-positive bacterial cell walls, which contain more negatively charged teichoic acids or lipoteichoic acids [102]. This increased negative charge enhanced the interaction between the bacterial cell wall and the positively charged N-CQDs, leading to more effective bacterial disruption. Therefore, this variability emphasizes the need to carefully consider both the physicochemical properties of CQDs and the structural characteristics of the target microorganisms when designing antimicrobial food-packaging materials.

## 5. Application of CQDs in Food Packaging

Over the past few years, the antioxidant and antimicrobial properties of CQDs have been explored across various sectors, including biomedicine, environmental remediation, and agriculture [50,86,104]. An emerging field of interest is the use of CQDs in food-packaging materials to improve the safety and shelf life of food products. CQDs, synthesized from sustainable biomass, possess the ability to neutralize ROS and inhibit microbial growth. These dual functionalities offer promising solutions for improving food preservation and promoting sustainable packaging. The incorporation of CQDs into food packaging represents a significant advancement in sustainable packaging by leveraging the biocompatibility, low toxicity, and renewable nature of CQD precursors [86,97].

CQDs are synthesized from various biomass sources, such as fruit and vegetable peels, leaves, and marine residues, using techniques such as hydrothermal processing, microwave-assisted synthesis, ultrasonication, and pyrolysis. These synthesis routes facilitate the production of CQDs with tailored surface functionalities, thereby enhancing their integration into biopolymer-based packaging materials. When integrated into active packaging, coatings, or intelligent systems, CQDs exhibit significant antioxidant properties by scavenging ROS, thereby preventing the oxidative spoilage of perishable foods, such as meat, fruits, and dairy products. Additionally, their antimicrobial properties, which are driven by the generation of ROS and the disruption of bacterial cell membranes, effectively inhibit the growth of spoilage-causing microorganisms. The multifunctionality of CQDs, acting as both antioxidants and antimicrobials, makes them efficient nanomaterials for extending the shelf life of perishable foods, promoting food safety, and minimizing waste in an environmentally sustainable manner [105].

Recently, various applications of CQDs in food packaging have been explored (Table 1). Yang et al. developed CQDs from coffee husks and incorporated them into CMC films [52]. These films displayed a scavenging efficiency of more than 95% in DPPH and ABTS assays, as well as strong antibacterial action against *L. monocytogenes* and *E. coli*, extending the freshness of fresh-cut apples by 7 d at 4 °C. Similarly, CQDs synthesized from radish residues and incorporated into a starch/chitosan film showed strong antioxidant activity, with scavenging efficacies of 93.8% for DPPH and 99.36% for ABTS, and effectively inhibited the growth of *Aeromonas sobria* and *Hafnia alvei*, thereby enhancing the preservation of salmon fillets [106]. In a study by Nazar et al., CQDs synthesized from Arabica coffee waste were incorporated into bio-composites made of purple sweet potato starch, chitosan, and Moringa leaf extract. The results demonstrated that the integration of CQDs into these polymer matrices not only enhanced the UV-barrier and optical properties of developed films but also improved their thermomechanical properties and antioxidant behavior. Importantly, the incorporation of CQDs did not adversely affect the biodegradability of the developed films, suggesting that CQDs can be integrated into biodegradable polymer systems without compromising their environmental sustainability [107]. These studies demonstrated the significant role of CQDs in enhancing the functional properties of biopolymer packaging materials. Hydrogel films, which are another advancement in food packaging, have shown substantial potential when combined with CQDs. For example, Zhang et al. developed a chitosan-based hydrogel film containing CQDs synthesized from glucose and l-tryptophan, which exhibited excellent antioxidant and antimicrobial properties. These hydrogel films effectively preserved strawberries and oranges for prolonged periods, confirming the functional attributes of the CQDs in preserving food freshness [108].

In addition, the use of CQDs in coatings applied to food surfaces or packaging has become a growing area of interest. These coatings provide barriers to gases and moisture while offering strong antioxidant and antimicrobial properties [115]. For instance, coatings with CQDs have shown considerable effectiveness in extending the shelf life of perishables, such as fruits, vegetables, and meat. A lemon coating with CQDs derived from ascorbic acid and chitosan incorporated into a CMC matrix demonstrated excellent antioxidant performance (88% DPPH and 100% ABTS scavenging) and bactericidal activity against *E. coli* and *L. monocytogenes*, preventing mold growth for 21 d [43]. Ezati et al. demonstrated the effectiveness of glucose-derived CQDs incorporated into chitosan/gelatin coatings in preventing mold on avocados and extending their shelf life by more than 14 d [24].

Within modern food-packaging systems, smart films not only protect food but also enable the real-time monitoring of food quality. CQDs with adjustable PL are used in these films to produce color changes or fluorescence based on environmental variations within the packaging. Wagh et al. developed gelatin-based smart films by integrating CQDs derived from biowaste pomace of blueberries [113]. These films exhibited antioxidant properties (scavenging efficiencies of 48.7% for DPPH and 54.4% for ABTS) and potent antibacterial activities against *L. monocytogenes* and *E. coli*. The smart films effectively indicated quality changes in real time, prolonging the shelf life of minced pork, fish, and shrimp by maintaining their freshness. These studies illustrate the versatility of CQDs as sustainable nanomaterials that significantly enhance the functional properties of packaging films while satisfying the growing demand for environmentally friendly and nontoxic food preservation solutions. The wide applicability of CQDs as active films, hydrogel films, coatings, or smart films demonstrates their increasing importance in the food-packaging sector. The combination of their antioxidant and antimicrobial properties, when sourced from various renewable carbon precursors, reflects their critical role in addressing modern food preservation challenges and supporting sustainable packaging practices [36,88].

## 6. Conclusions and Future Perspectives

Recently, there has been an increasing focus on sustainable development across various sectors, particularly in food packaging. The application of nanomaterials derived from diverse sustainable and nontoxic sources is a key focus in satisfying the demand for ecofriendly packaging solutions. In this context, CQDs have been used as novel and promising nanomaterials to fulfill the demands of functionality and sustainability. Owing to their remarkable antioxidant and antimicrobial activities, CQDs offer a promising solution for active and intelligent packaging by providing a sustainable alternative to toxic and expensive nanoparticles. CQDs synthesized from sustainable biomass sources, such as fruit peels, leaves, agricultural residues, and other organic materials, are highly attractive owing to their low toxicity, high biocompatibility, and environmentally friendly production methods. These nanomaterials can be synthesized using techniques such as hydrothermal, microwave-assisted, and pyrolysis methods, which not only facilitate scalable production but also allow for tailored surface functionalities that enhance integration into biopolymer matrices. Their application in active packaging systems is particularly significant because they effectively scavenge ROS, thereby preventing oxidative spoilage in perishable foods, such as fruits, dairy products, and meat. Additionally, the antimicrobial properties of CQDs, which are attributed to their ability to generate ROS and disrupt bacterial cell membranes, provide an additional defense against microorganisms that cause spoilage.

Although promising advances have been made, several obstacles must be overcome before CQDs can be widely adopted in food-packaging applications. One of the main challenges is scaling up the production of CQDs using lab-based methods, such as hydrothermal synthesis and pyrolysis. Industrial-scale production demands consistent quality, higher yields, and cost efficiency, which require further optimization. Moreover, ensuring that CQDs maintain a uniform size, surface chemistry, and photoluminescent characteristics is key to preserving their functionality in packaging materials. Another key concern is regulatory approval. Although CQDs are generally regarded as relatively safe and nontoxic, thorough toxicological assessments are necessary to ensure their safety, particularly as food-contacting materials. Regulatory organizations, such as the U.S. Food and Drug Administration and European Food Safety Authority, need to establish clear guidelines for the use of CQDs in food packaging. These assessments should also consider potential long-term environmental impacts, including CQD degradation, migration into food, and interactions with other packaging components. 

Future studies should focus on overcoming these challenges by improving green and scalable synthesis sources, such as agricultural waste or other renewable biomass sources, to lower production costs and environmental impacts. Scalable synthesis techniques can play a significant role in the transition of CQDs from laboratory research to broader commercial applications. Furthermore, advances in functionalization strategies, including doping CQDs with elements, such as nitrogen or sulfur, can enhance their antioxidant and antimicrobial effects, making them more suitable for use in biopolymer-based packaging. Additionally, the integration of CQDs into intelligent packaging systems has significant potential. The tunable PL of CQDs can be utilized for the real-time monitoring of food conditions, providing visual signals of spoilage or contamination. Moreover, the incorporation of CQDs into hydrogel films, edible coatings, and other advanced packaging systems has opened new avenues for creating sustainable, high-performance packaging solutions.

The integration of CQDs into biopolymer matrices represents a significant advancement in active food packaging. As ongoing studies continue to address the challenges of production scaling, ensuring regulatory compliance, and guaranteeing safety, CQDs-based packaging solutions hold immense potential for transforming the food-packaging sector. Their antioxidant and antimicrobial properties, coupled with their biocompatibility and eco-friendly synthesis approaches, make CQDs a novel approach for addressing modern food preservation needs while supporting global sustainability efforts.

## Figures and Tables

**Table 1:** Antioxidant and antimicrobial performance of CQDs derived from various biomass sources in food-packaging applications.

Packaging Type	CQDs Precursor	Antioxidant Activity	Antimicrobial Activity	Applications	References
Active film	Coffee husk waste	Exhibited more than 95% free radical scavenging efficiency in DPPH and ABTS assays	Demonstrated strong antibacterial action against *L. monocytogenes* and *E. coli*	CMC/CQDs composite film delayed the spoilage of fresh-cut apples, extending shelf life to 7 d at 4 °C	[52]
Active film	Radish residue	Demonstrated high scavenging efficiencies of 93.8% for DPPH and 99.36% for ABTS	Displayed marked antibacterial efficacy against *Aeromonas sobria* and *Hafnia alvei*	Enhanced starch/chitosan film with CQDs for the efficient preservation of salmon fillets	[106]
Active film	*Pseudomonas aeruginosa* and doping with sulfur	Showed significant antioxidant performance of S-doped CQDs with 85.07% DPPH and 94.89% ABTS scavenging	Reported high antibacterial effectiveness against *L. monocytogenes*	Inhibited microbial spoilage in minced pork and prevented discoloration in fresh-cut apples using PVA/S-CQDs composite films	[92]
Active film	Lemon (L-CQDs) and Onion (O-CQDS)	Reported approximately 80% and 90% DPPH free radical scavenging activity for L-CQDs and O-CQDs, respectively	Exhibited strong antifungal activity against *Rhizopus* sp., *Penicillium* sp., *Candida albicans*, *Aspergillus* sp., and *Botrytis cinerea*.	Minimized quality degradation and extended the shelf life of strawberries	[109]
Active film	Coffee grounds	Achieved high antioxidant performance with 98.2% scavenging of ABTS radicals and 78.8% scavenging of DPPH radicals	Exhibited effective antimicrobial potential, significantly inhibiting the growth of *E. coli* and *L. monocytogenes*	Prolonged the freshness of minced pork to 21 d at 4 °C with CQDs-incorporated cellulose nanofiber active packaging	[110]
Active film	Green tea	Demonstrated significant antioxidant potential in CQDs-incorporated chitosan/starch film, with 71.4% DPPH and 98% ABTS scavenging efficiencies	Showed substantial antibacterial activity in CQDs-chitosan/starch film, effective against *L. monocytogenes*, *E. coli*, and *S. aureus*	Extended the shelf life and maintained the freshness of minced pork wrapped in CQDs-infused chitosan/starch film	[25]
Active film	Banana	Displayed significant antioxidant efficiency in CQDs-infused PVA film, with 72.81% DPPH and 97.08% ABTS radical scavenging	Exhibited strong antibacterial efficacy in CQDs-based PVA film, inhibiting the growth of *S. aureus*, *B. subtilis*, and *E. coli*.	Effective as active packaging for extending the storage life of bananas, jujubes, and fried meatballs	[111]
Hydrogel film	Polyethylenimine and l-cysteine	Displayed notable antioxidant properties in CQDs-enhanced hydrogel film, with approximately 50% DPPH and 75% ABTS scavenging rates	Exhibited substantial antimicrobial potential in CQDs-infused hydrogel film, showing strong inhibition of *E. coli* and *L. monocytogenes*	Extended the freshness and storage quality of bananas for more than 5 d	[112]
Hydrogel film	Glucose and L-tryptophan	Exhibited substantial antioxidant potential in CQDs-integrated chitosan hydrogel, with 95.83% DPPH scavenging rate	Demonstrated strong antimicrobial effects in CQDs-infused chitosan hydrogel, inhibiting *E. coli* and *S. aureus*	Enhanced the freshness of strawberries for over 5 d and oranges for 20 d under ambient conditions	[108]
Coating	Ascorbic acid and chitosan	Demonstrated high antioxidant performance in CQDs-CMC film, with 88% DPPH and 100% ABTS scavenging efficiencies	Demonstrated a significant bactericidal effect on *E. coli* and *L. monocytogenes*	Lemons coated with CMC/CQD film maintained excellent appearance with no mold growth after 21 d of storage	[43]
Coating	Glucose	Displayed significant antioxidant performance in CQDs-infused chitosan/gelatin film solution, with more than 95% scavenging efficiency for DPPH and ABTS	Showed potent antimicrobial effects in CQDs-infused chitosan/gelatin films against *L. monocytogenes*, *E. coli*, *A. flavus*, and *C. orbiculare*	Inhibited mold formation on avocados and extended their storage life by more than 14 d using CQDs-enhanced chitosan/gelatin film	[24]
Smart film	Biowaste pomace of blueberries	Demonstrated scavenging activity with 48.7% for DPPH and 54.4% for ABTS	Showed effective antibacterial properties against *L. monocytogenes* and *E. coli*	CQDs-based smart film with anthocyanin and gelatin, effectively extending and monitoring the shelf life of minced pork, fish, and shrimp	[113]
Smart active double-layer film	Barley bran	Displayed strong antioxidant properties in CQDs-incorporated film, with approximately 80% scavenging for DPPH and 90% for ABTS	Demonstrated potent antibacterial activity targeting *S. aureus* and *E. coli*	Implemented as a smart active double-layer film, extending *Ictalurus punctatus* fish shelf life by 2 d with integrated freshness monitoring	[114]
